# Coffee Consumption Is Associated With Later Age‐at‐Onset of Parkinson's Disease

**DOI:** 10.1002/acn3.70076

**Published:** 2025-06-12

**Authors:** Dariia Kuzovenkova, Lang Liu, Ziv Gan‐Or, Konstantin Senkevich

**Affiliations:** ^1^ Institute of Applied Computer Science ITMO University Saint Petersburg Russia; ^2^ The Neuro (Montreal Neurological Institute‐Hospital), McGill University Montreal Quebec Canada; ^3^ Department of Neurology and Neurosurgery McGill University Montreal Quebec Canada; ^4^ Department of Human Genetics McGill University Montreal Quebec Canada; ^5^ Division of Medical Genetics, Department of Specialized Medicine McGill University Health Centre Montreal Quebec Canada

**Keywords:** age of onset, coffee, Parkinson's disease

## Abstract

Observation studies suggest that coffee consumption may lower the risk and delay the age‐at‐onset (AAO) of Parkinson's disease (PD). The aim of this study was to explore the causal relationship and genetic association between coffee consumption and the AAO, risk, and progression of PD. Using Mendelian randomization, we identified a significant association between coffee consumption and delayed PD AAO (IVW: OR, 1.91; 95% CI 1.53–2.38; *p* = 8.072e‐09), but no causal association or genetic correlation with PD risk or progression. Our findings suggest a potential causal effect of higher coffee consumption on PD AAO, with no evidence of an association with PD risk or progression.

## 1 Introduction

Prospective studies over the past two decades have consistently associated caffeine consumption with a reduced risk of Parkinson's disease (PD) [1]. Additionally, lower levels of caffeine and its metabolites have been observed in PD patients compared to controls [2]. Although a clinical trial has not demonstrated significant symptomatic benefits of caffeine intake for PD [3], its potential to delay motor symptom onset may create an apparent protective effect.

The potential protective effects of caffeine in PD may involve multiple mechanisms, including antagonism of adenosine receptors and dopaminergic modulation [4]. Observational studies further support protective effects, demonstrating a later age at onset (AAO) of PD in analyses of coffee and tea consumption [5, 6, 7, 8]. However, studies examining caffeine consumption and PD progression have produced conflicting results [9, 10, 11, 12]. Despite previous Mendelian randomization (MR) studies failing to identify a causal link between coffee consumption and PD risk [13, 14], the effect on AAO and clinical progression remains underexplored. In addition, no strong genetic link between caffeine consumption and sporadic PD that would explain the protective association has been demonstrated to date.

To address the gaps in understanding the relationship between caffeine consumption and PD, we aimed to investigate the genetic and causal associations between the amount of coffee intake and PD AAO, risk, and progression. Using MR and genetic correlation, we assessed the relationship between coffee consumption and PD outcomes. We further constructed a polygenic risk score (PRS) for coffee consumption and studied the association with PD risk and AAO.

## 2 Methods

### 2.1 Study Population

We used publicly available genome‐wide association study (GWAS) data to explore the genetic and causal relationships between caffeine consumption and PD AAO, risk, and progression. Independent GWAS significant single nucleotide polymorphisms (SNPs) associated with caffeine consumption were obtained from the UK Biobank GWAS data (Data field ID 1498) [15] (Table 1). As an outcome, we used the largest available GWAS statistics on PD risk with 27,693 participants of European ancestry (PD cases = 15,056; controls = 12,637) [16]. UK Biobank data was excluded from it to prevent overlapping samples that could introduce bias. We also used GWAS results for PD AAO in the form of a continuous variable with 17,996 PD patients of European ancestry [17]. To examine coffee consumption's effects on PD symptom progression (UPDRS Part III), cognitive decline (MoCA, MMSE), insomnia, and hyposmia, we used publicly available GWAS summary statistics data for these traits [18]. For the genetic correlation analysis, we used a caffeine consumption GWAS for which complete summary statistics was available [19].

**TABLE 1:** GWAS summary statistics utilized in the MR analysis.

Studied trait	Study	Sample size, *N*	Type of trait
*Mendelian randomization exposure*			
Coffee consumption	PMID: 31412118 [9]	408,191	Continuous
*Mendelian randomization outcome*			
PD risk	PMID: 31701892 [10]	Cases = 15,056; controls = 12,637	Binary
PD AAO	PMID: 30957308 [11]	17,996	Continuous
UPDRS3	PMID: 31505070 [12]	1398	Continuous
MMSE		1329	Continuous
MoCA		1000	Continuous
Hyposmia		1027	Continuous
Sleep		1136	Continuous
*Genetic correlation*			
Coffee consumption	PMID: 33287642 [13]	362,316	Continuous

Abbreviations: AAO, age at onset; MMSE, Mini Mental State Examination; MoCA, Montreal Cognitive Assessment; PD, Parkinson disease; UPDRS3, unified Parkinson's disease rating scale part 3.

### 2.2 Mendelian Randomization and Genetic Correlation

MR analysis was used to assess the causal relationship between caffeine consumption and PD traits, relying on three key assumptions: relevance, independence, and exclusion restriction [20]. First, to ensure the relevance assumption, we selected SNPs significantly associated with caffeine consumption (*p* < 5 × 10^−8^) as instrumental variables (IVs) and confirmed instrument strength using F‐statistics (threshold ≥ 10). Second, to mitigate potential violations of the independence assumption, we applied linkage disequilibrium clumping (*r* ^2^ < 0.001, 10,000 kb window) and used non‐overlapping GWAS datasets for exposure and outcome as mentioned above. Third, to address the exclusion restriction assumption, which assumes that the instruments influence the outcome only through the exposure—we performed multiple sensitivity analyses. We applied Steiger filtering to remove genetic variants that explained more variance in the outcome trait than in the exposure, helping confirm the correct causal direction. Pleiotropic SNPs were identified and excluded using MR‐PRESSO [21]. The inverse variance‐weighted (IVW) method was the primary MR approach, with MR‐Egger and weighted median estimates as sensitivity analyses [22]. Directional pleiotropy was assessed using the MR‐Egger intercept test, and heterogeneity was evaluated with Cochran's Q test. Genetic correlation between caffeine consumption and PD traits was calculated using linkage disequilibrium score regression [23].

### 2.3 Polygenic Risk Score

We evaluated the cumulative genetic contribution of caffeine consumption–associated SNPs to PD risk by constructing a PRS from genome‐wide significant variants associated with caffeine intake [15]. Linkage disequilibrium (LD) clumping (*r* ^2^ < 0.1, 250 kb window) was applied, and scores were adjusted for age, sex, and principal components. The PRS was then tested across seven independent cohorts for both PD risk and AAO (Table S1), followed by a meta‐analysis of the combined results.

## 3 Results

**FIGURE 1:**
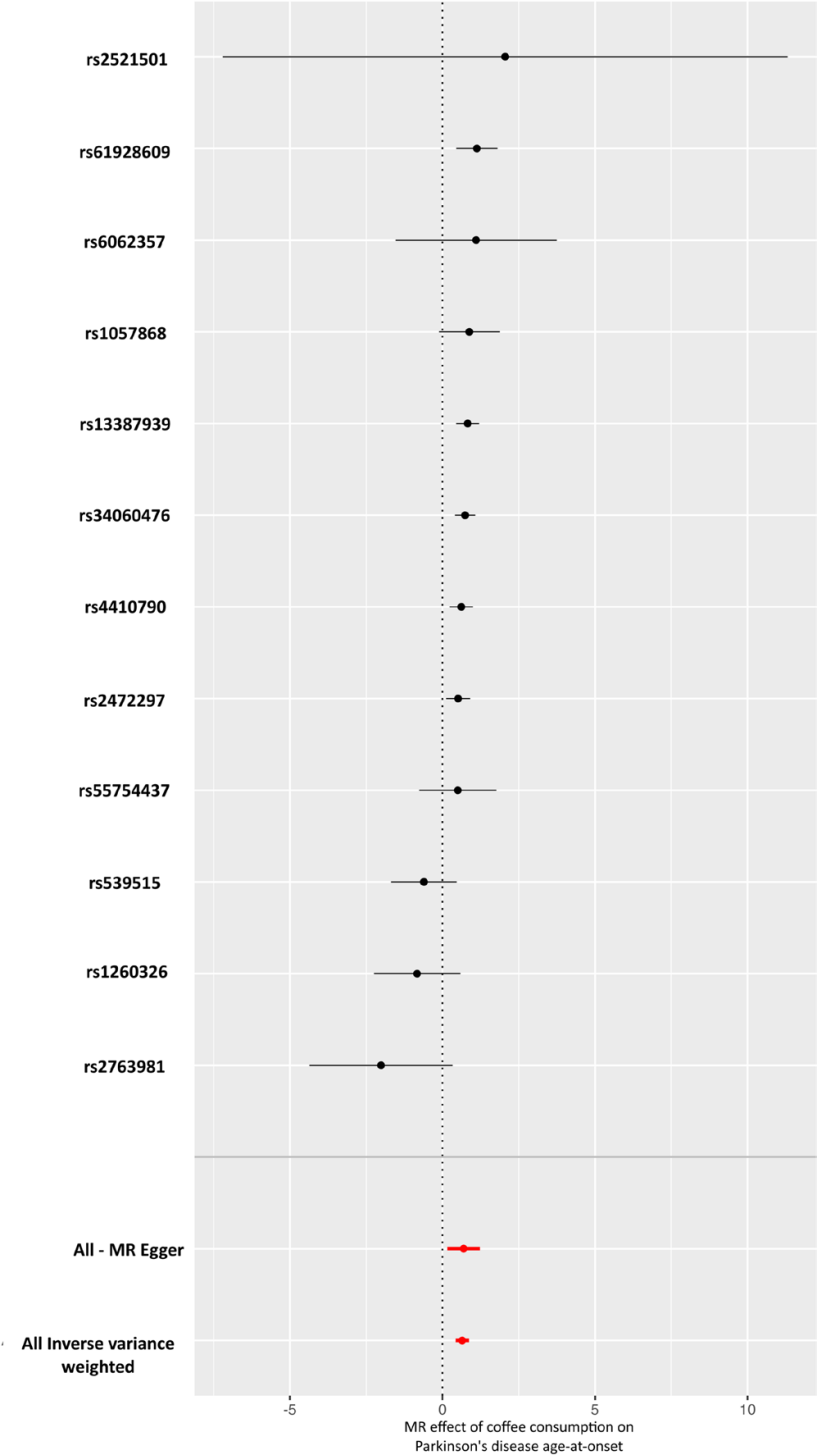
Forest plot of the MR analysis for the causal association between coffee consumption and the age at onset (AAO) of Parkinson's disease (PD). Each point on the *y*‐axis corresponds to a single nucleotide polymorphism (SNP) used as an instrumental variable (IV). The *x*‐axis shows the effect size (beta) for each SNP, with horizontal lines depicting 95% confidence intervals. In this scale, larger effect sizes indicate a later AAO (i.e., a delay in PD onset). The bottom two points display the overall MR‐Egger and inverse variance–weighted (IVW) estimates, respectively, summarizing the combined effect of all IVs.

We performed MR analysis of coffee consumption and PD AAO using 28 SNPs as IVs. The initial results showed no causal association (IVW: OR, 2.260; 95% CI, 0.568–8.989; *p* = 0.247). However, MR‐PRESSO identified 16 pleiotropic SNPs (Table S2), which were excluded from a subsequent analysis. After removing pleiotropic SNPs, MR analysis with the remaining 12 SNPs revealed a strong causal association between coffee consumption and PD AAO (IVW: OR, 1.909; 95% CI, 1.532–2.377; *p* = 8.072e‐09; Figure 1; Table 2). Because AAO is a continuous outcome, OR > 1 indicates that greater coffee consumption is causally linked to a later onset of PD. Sensitivity analyses confirmed the robustness of these findings. No residual pleiotropy was detected (MR‐PRESSO: *p* = 0.202; Egger: *p* = 0.851), and heterogeneity tests showed no evidence of inconsistency (Cochran's Q for IVW: *p* = 0.074; Table S3).

**TABLE 2:** Mendelian randomization estimates of genetically predicted coffee consumption on Parkinson's disease risk, AAO, and progression.

Outcome	*N*, SNPs	Inverse variance weighted				MR Egger			
		OR	L_CI95	U_CI95	*p*	OR	L_CI95	U_CI95	*p*
PD AAO	12	1.909	1.532	2.377	8.072e‐09	2.202	1.169	3.430	0.030
PD risk	17	0.734	0.472	1.142	0.694	0.590	0.273	1.275	0.933
UPDRS3	21	0.951	0.714	1.267	0.934	0.820	0.496	1.356	0.933
MMSE	4	0.921	0.522	1.625	0.776	0.970	0.307	3.067	0.964
MoCa	7	1.037	0.233	4.614	0.962	0.902	0.051	15.666	0.947
Hyposomia	21	1.564	0.571	4.281	0.694	2.294	0.388	13.579	0.933
Sleep	7	1.484	0.523	4.206	0.458	1.940	0.314	11.994	0.508

Abbreviations: AAO, age at onset; L_CI95, low 95% confidence interval; MMSE, Mini Mental State Examination; MoCA, Montreal Cognitive Assessment; OR, odds ratio; PD, Parkinson's disease; UPDRS3, unified Parkinson's disease rating scale part 3; U_CI95, upper 95% confidence interval.

We then evaluated whether coffee consumption influences the risk or clinical progression of PD, including motor (UPDRS3), cognitive (MMSE and MoCA), and non‐motor features (hyposmia and sleep). Across all analyses, we observed no significant causal effects of coffee consumption (Table 2). Sensitivity analyses further confirmed the robustness of these null results (Table S3).

Finally, no significant genetic correlation between coffee consumption and PD risk or AAO was observed (Table S4). PRS for coffee consumption also did not reveal significant associations with PD risk (OR = 1.02, 95% CI: 0.993–1.048, *p* = 0.147) or AAO (OR = 0.083, 95% CI: −0.108 to 0.274, *p* = 0.397; Table S5).

## 4 Discussion

Our findings indicate a potential protective relationship between coffee consumption and PD onset, suggesting that greater coffee intake may delay the emergence of clinical symptoms without altering overall disease risk or progression. After addressing pleiotropy in the MR analysis, we observed a robust association with later AAO, whereas neither MR, PRS, nor genetic correlation analyses supported an effect on PD risk. These results align with previous reports linking coffee consumption to a delayed symptomatic onset of PD [5, 24] and further refine our understanding of coffee consumption's role in PD, pointing toward mechanisms that modulate symptom onset rather than disease susceptibility.

One hypothesis is that the antagonistic effect of coffee on adenosine A2A receptors may delay the emergence of overt motor symptoms, possibly by masking early clinical features and thereby leading to delayed diagnosis rather than a true neuroprotective effect. Notably, we identified several pleiotropic variants related to traits such as energy metabolism and smoking habits—one of the SNPs was located near the *ADORA2A* receptor gene, yet none of these variants independently influence PD risk or AAO. Meanwhile, istradefylline, a selective adenosine A2A receptor antagonist, has been approved in several countries and shown efficacy in improving motor symptoms of PD [25]. Earlier MR studies have likewise failed to establish a causal association between coffee intake and PD risk [13, 14], suggesting that the effect of coffee may indeed be selective for AAO.

Further research in prodromal cohorts, particularly those with REM sleep behavior disorder (RBD), is needed to clarify whether coffee consumption can delay or modify progression to PD. Although some evidence does not support a protective effect on RBD phenoconversion [26, 27], other findings suggest that RBD patients who subsequently developed parkinsonism rather than dementia consumed more caffeine [26]. Meanwhile, one of the few studies conducted in genetically stratified cohorts demonstrated lower caffeine concentrations and its metabolites among carriers of *LRRK2* variants [28], which is notable given that *LRRK2*‐PD is typically characterized by a lower prevalence of RBD and reduced dementia rates compared with sporadic or GBA‐associated PD [29].

We acknowledge several limitations in our analysis. First, our study focused on individuals of European descent, which reflects the largest available GWAS data but limits the generalizability of our findings to other populations. Second, the available GWASs for PD progression phenotypes are relatively small and may be underpowered to detect an association. Third, even after excluding all detected pleiotropic SNPs from MR, residual pleiotropy cannot be completely ruled out. Furthermore, the protective signal may predominantly reflect specific genetic subtypes of PD, emphasizing the need to stratify PD by genetic subtypes in future research. Finally, we did not investigate potential sex‐specific effects or interactions with specific therapies, which could influence disease onset and progression.

Overall, our findings suggest that coffee consumption appears to delay PD onset rather than reducing overall disease risk or modifying disease phenotype. Importantly, these results reflect the effect of genetically predicted coffee consumption on PD AAO as captured by MR and thus may not directly translate to increased coffee intake in everyday dietary practice. Future research should focus on validating these observations in broader and genetically diverse populations, ultimately aiming to clarify the mechanistic basis and therapeutic potential of coffee in PD.

## Author Contributions

D.K. and K.S.: contributed to the conception and design of the study. D.K., L.L., Z.G.‐O., and K.S.: contributed to the acquisition, analysis of data, and contributed to drafting the text and preparing the figures.

## Conflicts of Interest

The authors declare no conflicts of interest.

## Supporting information


Table S1.

## Data Availability

The code used for the analysis is available on GitHub: https://github.com/senkkon/coffee_PD/. Summary statistics used in the analysis are publicly available. The cohorts used for PRS analysis are listed in Table S1 and in Acknowledgements. Access to these cohorts is restricted to qualified researchers and can be requested through their respective data access portals or governing bodies.
